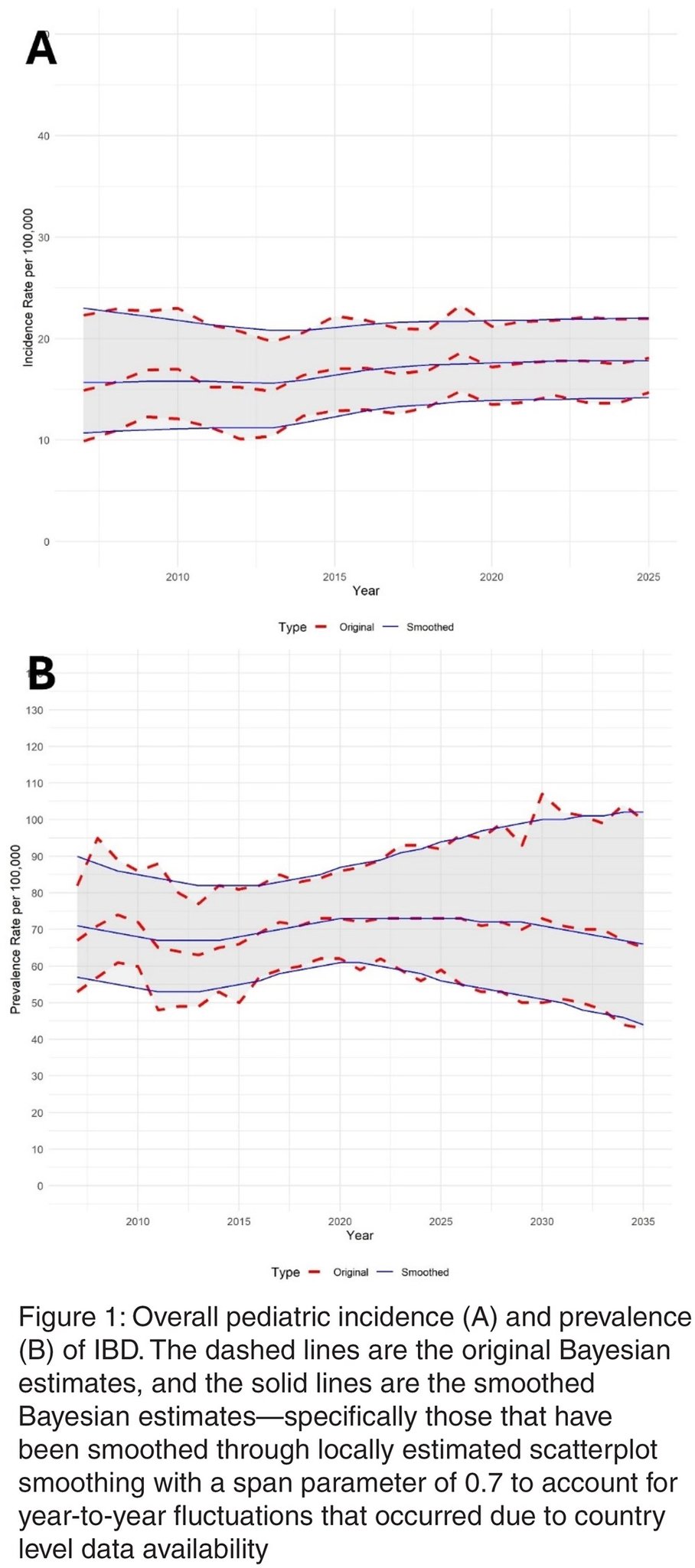# Poster Session II – Poster of Distinction II - A210 THE INCIDENCE AND PREVALENCE OF PEDIATRIC INFLAMMATORY BOWEL DISEASE IN NINE NATIONS ACROSS THE GLOBE: AN ANALYSIS OF THE 3^RD^ EPIDEMIOLOGIC STAGE

**DOI:** 10.1093/jcag/gwaf042.210

**Published:** 2026-02-13

**Authors:** S Coward, J W Windsor, L Hracs, J Gorospe, S Okabayashi, E Kuenzig, E Benchimol, G G Kaplan

**Affiliations:** University of Calgary, Calgary, AB, Canada; University of Calgary, Calgary, AB, Canada; University of Calgary, Calgary, AB, Canada; University of Calgary, Calgary, AB, Canada; University of Calgary, Calgary, AB, Canada; Western University, London, ON, Canada; The Hospital for Sick Children, Toronto, ON, Canada; University of Calgary, Calgary, AB, Canada

## Abstract

**Background:**

The prevalence of pediatric (<18 years) inflammatory bowel disease (IBD) has steadily increased in North America, Europe, and Oceania, driven by a phenomenon known as compounding prevalence or epidemiologic stage 3. However, comprehensive analyses of population-based pediatric epidemiologic surveillance data from stage 3 countries remains limited.

**Aims:**

To estimate the incidence and prevalence of pediatric IBD in 2025 and 2035 among countries in the third epidemiologic stage by forecasting historic, longitudinal data.

**Methods:**

Population-based administrative data on the incidence and/or prevalence of pediatric IBD were acquired from Canada (2002–2014), Catalonia (2011–2020), Denmark (1990–2017), Hungary [Veszprém] (2002–2018), Israel (2005–2020), New Zealand [Canterbury] (incidence only: 2018–2023), Scotland [Lothian] (2009–2017), and Sweden (1990–2014). Rates were sex standardized to the matching year of the Canadian population. Incidence and prevalence (per 100,000) of individual countries were forecasted to 2025 (incidence) and 2035 (prevalence) using autoregressive integrated moving average models (ARIMA), with associated 95% prediction intervals (PI). Overall pediatric forecasted rates, with 95% credible intervals (CrI), were calculated using Bayesian analysis with a multilevel Poisson model that included random intercepts for each country. Average annual percentage change (AAPC), with 95% confidence intervals (CI), were calculated using Poisson or negative binomial models.

**Results:**

Incidence and prevalence of pediatric IBD are forecasted remain stable with AAPCs of 0.93% (95%CI: −1.08, 2.98) and 0.07% (95%CI: −0.45, 0.59), respectively. In 2025, incidence is forecasted to be of 17.8 per 100,000 (95% CrI: 14.2, 22.0) and prevalence 73 per 100,000 (95%CrI: 56, 94). By 2035 the prevalence is forecasted to be 66 per 100,000 (95%CrI: 44, 102).

**Conclusions:**

The incidence of pediatric IBD in stage 3 countries is forecast to have stabilized at 17.8 cases per 100,000 population. Prevalence is projected to remain stable over the next decade as affected individuals transition from pediatric to adult gastroenterology care. These data are instrumental for healthcare planning, informing infrastructure development, resource allocation, and workforce needs to effectively manage pediatric IBD in the coming years.

**Funding Agencies:**

The Leona M. and Harry B. Helmsley Charitable Trust